# Supplementary material for: Monoacylglycerol lipase regulates cannabinoid receptor 2-dependent macrophage activation and cancer progression
Source: Nat Commun. 2018 Jul 3;9:2574. doi: 10.1038/s41467-018-04999-8 (PMC6030061; doi:10.1038/s41467-018-04999-8)
Supplement: Supplementary file 1 — Supplementary Information [file 41467_2018_4999_MOESM1_ESM.pdf]

Monoacylglycerol lipase regulates cannabinoid receptor 2-dependent macrophage activation and cancer progression

Wei Xiang[1,#], Rongchen Shi[1,#], Xia Kang[1,#], Xuan Zhang[2,#], Peng Chen[3], Lili Zhang[1], Along Hou[1], Rui Wang[1], Yuanyin Zhao[1], Kun Zhao[1], Yingzhe Liu[1], Yue Ma[1], Huan Luo[1], Shenglan Shang[1], Jinyu Zhang[4], Fengtian He[1], Songtao Yu[2], Lixia Gan[1], Chunmeng Shi[5], Yongsheng Li[6,*], Wei Yang[7,*], Houjie Liang[2,*] Hongming Miao[1,*]

[1]Department of Biochemistry and Molecular Biology, Third Military Medical University, Chongqing 400038, China

[2]Department of Oncology, Southwest Hospital, Third Military Medical University, Chongqing 400038, China

[3]Department of General Surgery, PLA 324 Hospital, Chongqing 400020, China.

[4]National Engineering Research Center of Immunological Products, Third Military Medical University, Chongqing 400038, China

[5]Institute of Combined Injury, State Key Laboratory of Trauma, Burns and Combined Injury, Third Military Medical University, Chongqing 400038, China

[6]Clinical Medicine Research Center & Institute of Cancer, Xinqiao Hospital, Third Military Medical University, Chongqing 400037, China

[7]Department of Pathology, School of Basic Medical Sciences & Nanfang Hospital, Southern Medical University, Guangzhou 510515, China

[#]Co-first author

*Correspondence:

hongmingmiao@sina.com (H.M.M.), yli@tmmu.edu.cn(Y.S.L.), yangwei@sibcb.ac.cn (W.Y.), lianghoujie@sina.com (H.J.L.)

Supplementary Figures and Figure Legends

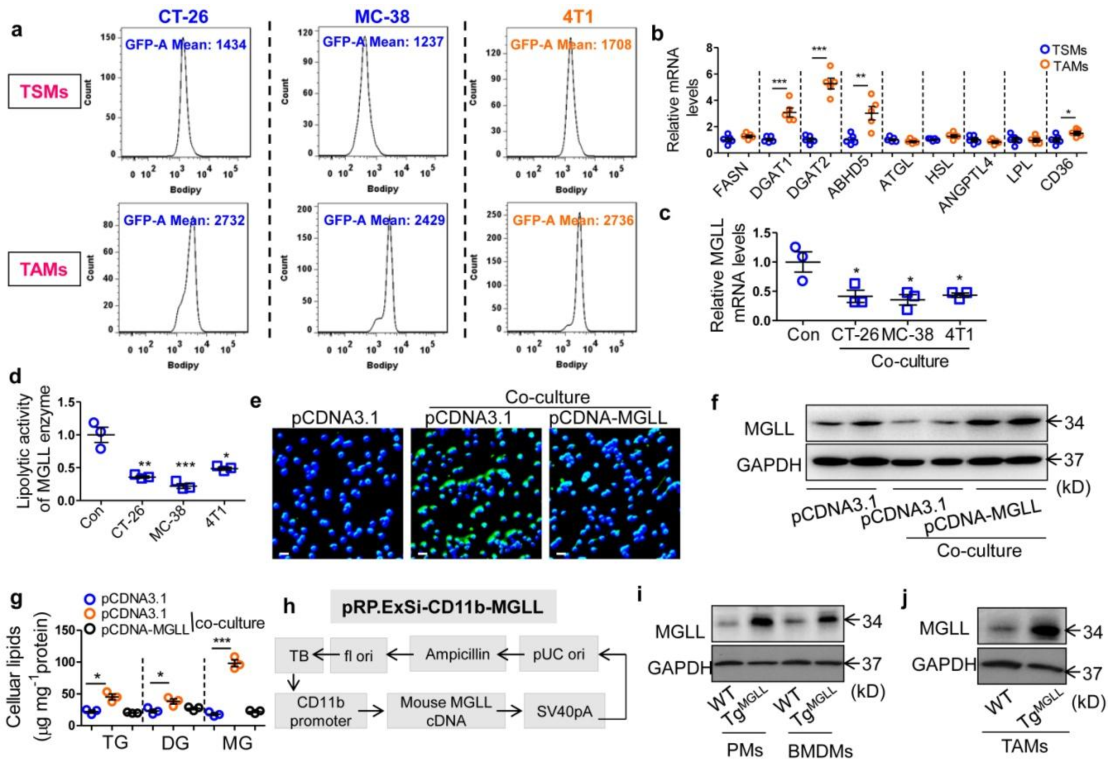

Supplementary Figure 1. MGLL in tumor-associated macrophages regulates lipid accumulation. Related to Figure 1.

a, The lipid levels in TSMs and TAMs. Six-week-old mice were subcutaneously injected with CT-26, MC-38 or 4-T1 cells. Two weeks later, spleen macrophages from tumor-bearing mice (TSMs) and TAMs were isolated for lipid staining with bodipy. The Geometric mean fluorescence intensity (MFI) of Bodipy in each group was measured. The tested sample in each group was pooled from 5 individual ones.

b, Expression of lipid metabolism-related enzymes in TSMs and TAMs. The TSMs and TAMs were obtained from mice that were inoculated with MC-38 tumors for 2 weeks. mRNA levels of FASN, DGAT1, DGAT2, ABHD5, ATGL, HSL, ANGPTL4, LPL and CD36 were measured by real-time PCR (n=5, *P<0.05, **P<0.01, ***P<0.005).

c, Murine peritoneal macrophages (PMs) were co-cultivated with CT-26, MC-38 or 4-T1 cells for 48 h. Then, the mRNA levels of MGLL in macrophages were measured by real-time PCR (n=3, *P<0.05).

d, Lipolytic activity of MGLL enzyme in the PMs described in (**c**) (n=3, *P<0.05,

**P<0.01, ***P<0.005).

e, MGLL overexpression blocked MC-38 cell-induced lipid accumulation in macrophage-like cells. Raw264.7 cells were transfected with pCDNA3.1 or pCDNA-MGLL, co-cultivated with MC-38 cells for 48 h and then stained with Bodipy (Green). The nucleus was visualized by DAPI staining (Blue). Representative images are displayed. Scale bars, 40 μm

f, Immunoblotting assays of MGLL in Raw264.7 cells described in (**e**).

g, Cellular lipid assays of the Raw264.7 cells described in (**e**) (n=3, *P<0.05, ***P<0.005).

h, The structure diagram of the plasmid used for the macrophage-specific MGLL transgene (Tg^{MGLL}).

i, Immunoblotting assays of MGLL in PMs or BMDMs from WT or Tg^{MGLL} mice.

j, Immunoblotting assays of MGLL in the TAMs from WT or Tg^{MGLL} mice bearing MC-38 tumors.

Data in panel (**b**), (**c**), (**d**) and (**g**) show the means±s.e.ms. and are analyzed by Student's *t*-test.

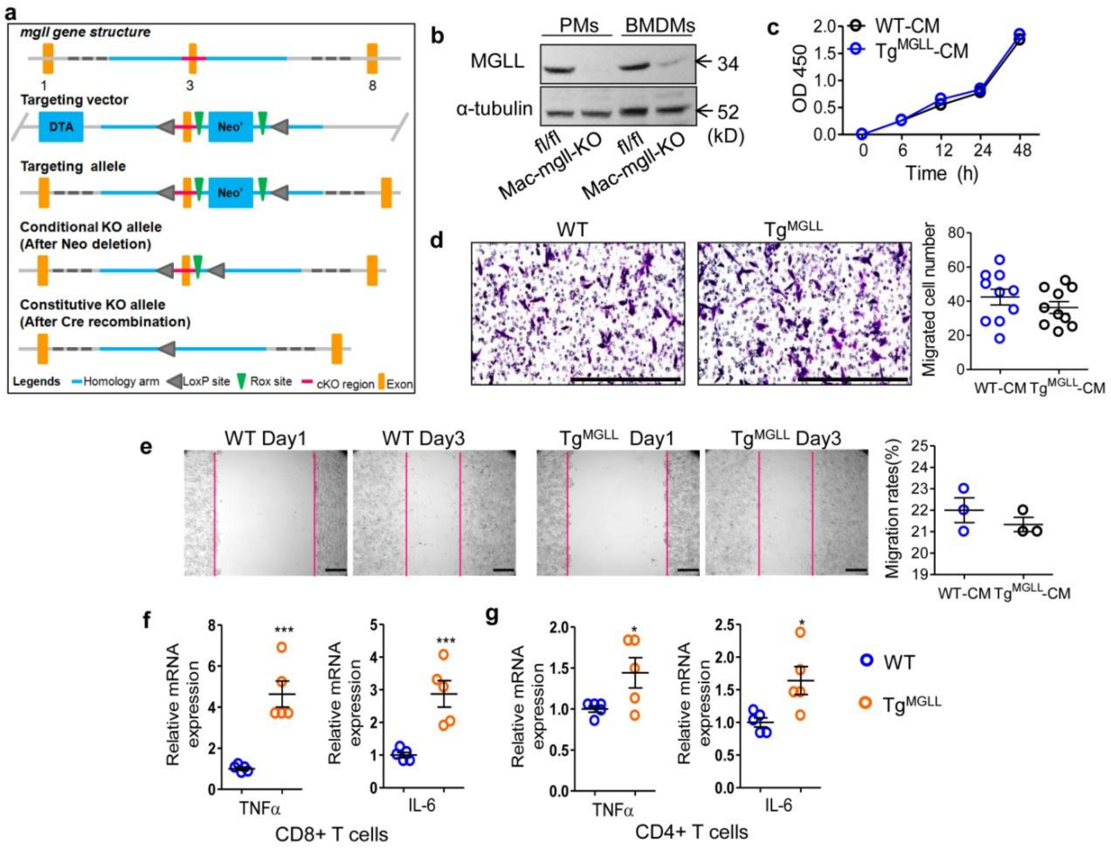

Supplementary Figure 2. Macrophage MGLL doesn't affect tumor progression in vitro. Related to Figure 2.

a, Overview of the targeting strategy in conditional knockout of the *mgll* gene in mice.

b, Immunoblotting assays of MGLL in PMs and BMDMs from fl/fl or Mac-mgll-KO mice.

c, Cell viability of MC-38 cells treated with CM from WT or Tg^{MGLL} peritoneal macrophages. The data represent the means±s.e.ms. (n=5, Student's *t*-test).

d-e, The migration activity of MC-38 cells induced by the CM from WT or Tg^{MGLL} macrophages was measured using transwell migration assays (**d**) or scratch tests (**e**). Representative images were displayed. The migrated cells or migration rates were calculated. The data represent the means±s.e.ms. (n=10 in transwell migration assays; n=3 in scratch tests; Student's *t*-test). Scale bars, 400 μm

f-g, mRNA levels of IFNγ in CD8+ (**f**) or CD4+ (**g**) T cells that were isolated from the MC-38 tumors inoculated subcutaneously in WT or Tg^{MGLL} mice for 3 weeks. The data represent the means±s.e.ms. (n=5, *P<0.05, ***P<0.005, Student's *t*-test).

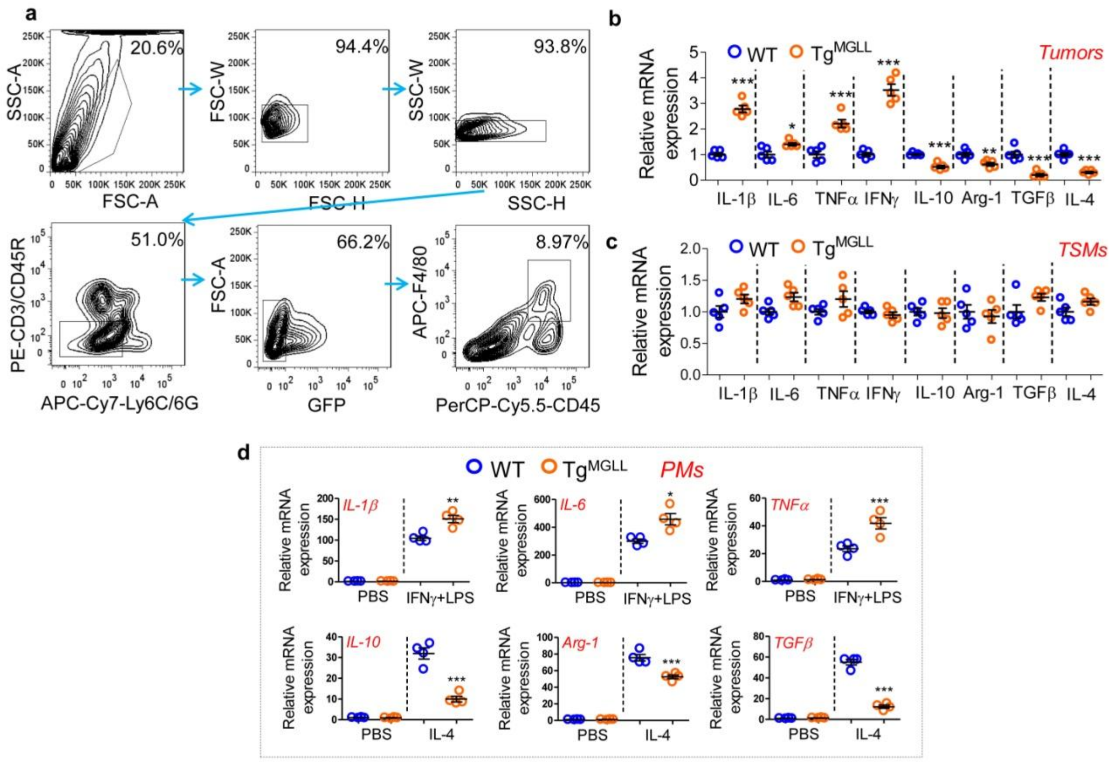

Supplementary Figure 3. Macrophage MGLL suppresses tumor growth by activating CD8+ T cells. Related to Figure 3.

a, FACS gating strategy for TAMs. TAMs were assessed as CD3-CD45R-Gr1-GFP-CD45+F4/80+. The inoculated MC-38 cells were stably marked with GFP.

b, mRNA expression of cytokines in MC-38 tumors, which were inoculated subcutaneously in WT or Tg^{MGLL} mice for 3 weeks (n=5, *P<0.05, **P<0.01, ***P<0.005).

c, mRNA expression of cytokines in spleen macrophages from the MC-38 tumor-bearing WT or Tg^{MGLL} mice. TSMs, spleen macrophages in the tumor-bearing mice (n=5).

d, mRNA levels of cytokines in the WT or Tg^{MGLL} peritoneal macrophages treated with IFNγ (10 ng ml^{-1}) + LPS (100 ng ml^{-1}) or IL-4 (10 ng ml^{-1}) for 6 h and then subjected to real-time PCR assays (n=4, *P<0.05, **P<0.01, ***P<0.005).

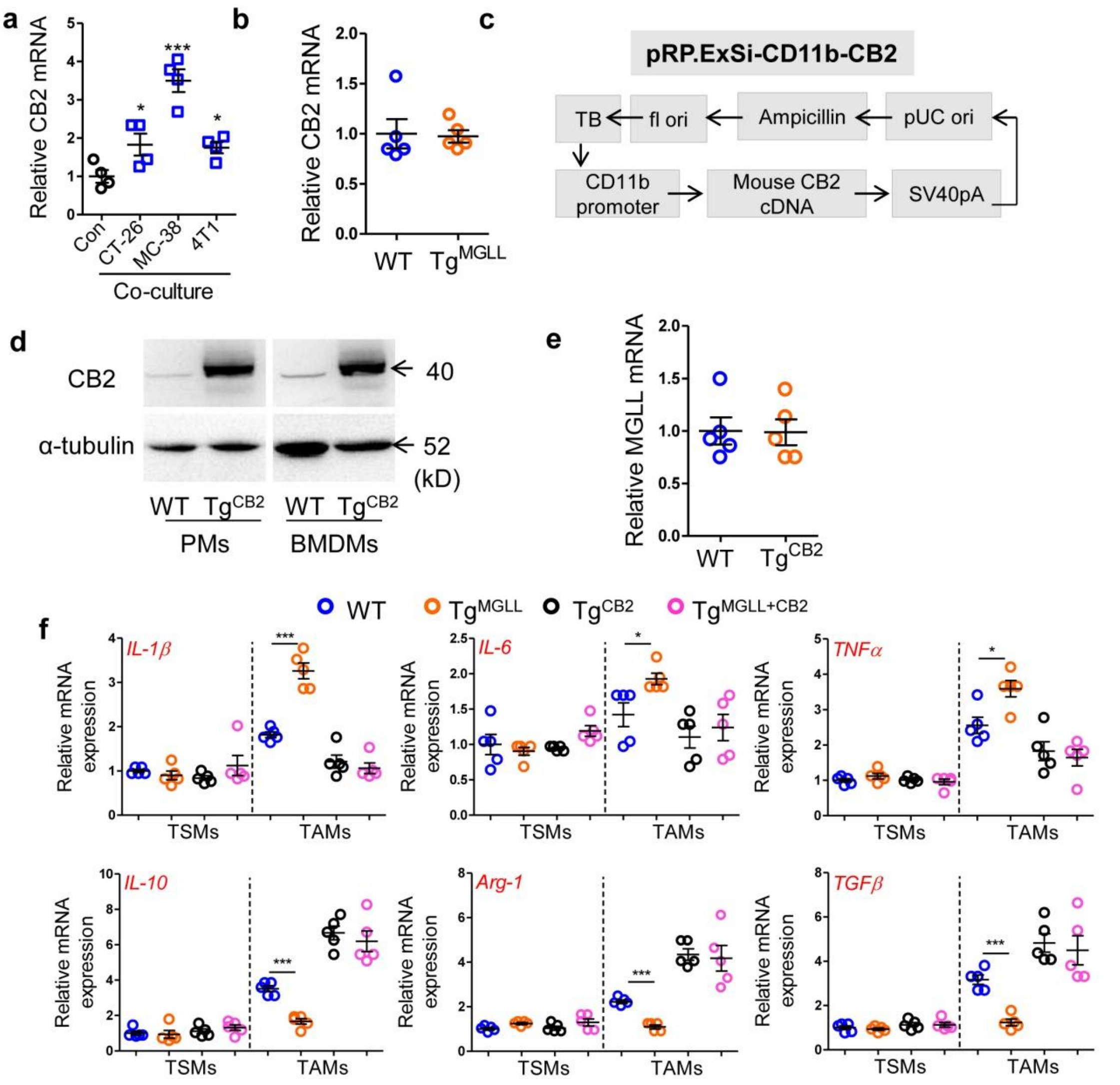

Supplementary Figure 4. MGLL regulates macrophage activation via CB2. Related to Figures 4-5.

a, Relative CB2 mRNA expression in peritoneal macrophages, which were co-cultivated with CT-26, MC-38 or 4-T1 cells for 48 h (n=4, *P<0.05, ***P<0.005).

b, Relative mRNA levels of CB2 in TAMs from MC-38 tumor-bearing WT or Tg^{MGLL} mice (n=5).

c, Structure diagram of the construct used for the macrophage-specific transgene of CB2 (Tg^{CB2}).

d, Immunoblotting assays of CB2 in PMs and BMDMs from WT and Tg^{CB2} mice.

e, Relative mRNA levels of MGLL in TAMs from MC-38 tumor-bearing WT or Tg^{CB2} mice (n=5).

f, Relative mRNA expression of cytokines in TAMs from the WT, Tg^{MGLL}, Tg^{CB2}

and Tg$^{MGLL+CB2}$ mice bearing MC-38 tumors for 3 weeks (n=5, *P<0.05, ***P<0.005).

Data in panel (**a**-**b**) and (**e**-**f**) represent means±s.e.ms. and are analyzed by Student's *t*-test.

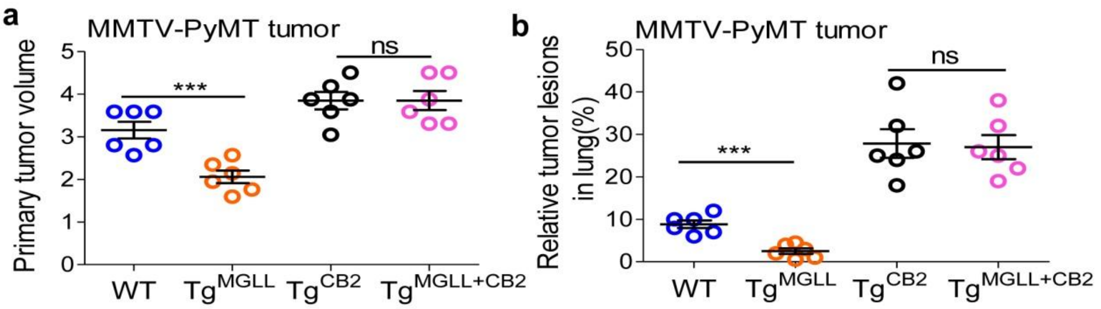

Supplementary Figure 5. Macrophage MGLL-CB2 axis regulates tumor progression in a genetic breast cancer model. Related to Figure 6.

a, Volume of the primary breast tumors in the WT, Tg^{MGLL}, Tg^{CB2} or $Tg^{MGLL+CB2}$ MMTV-PyMT mice. Data are means±s.e.ms. (n=6, ***P<0.005, Student's *t*-test; ns, not significant).

b, Tumor lesions in lungs from the WT, Tg^{MGLL}, Tg^{CB2} or $Tg^{MGLL+CB2}$ MMTV-PyMT mice. Data are means±s.e.ms. (n=6, ***P<0.005, Student's t-test; ns, not significant).

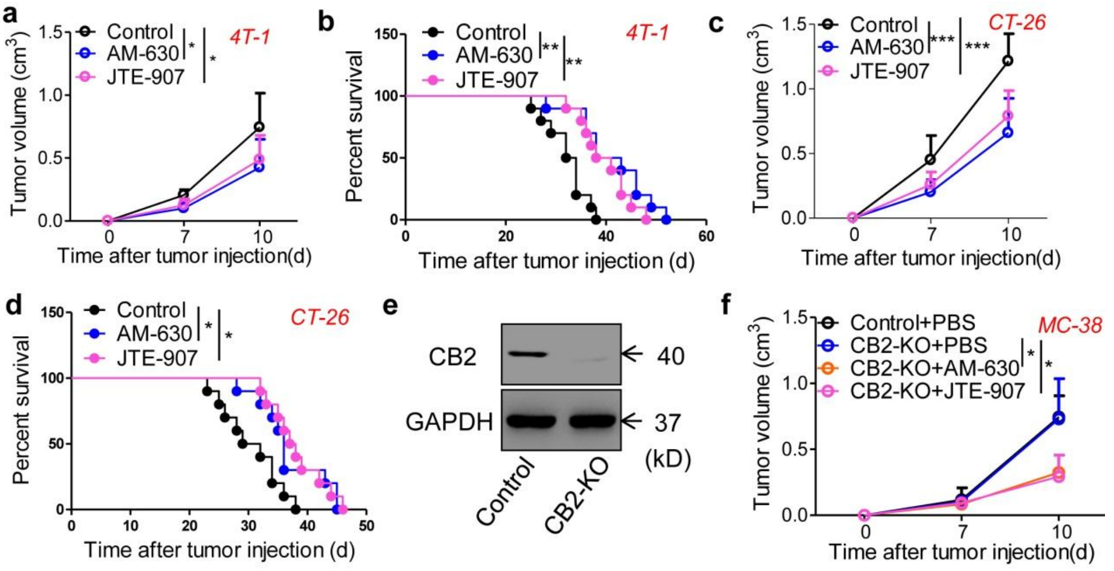

Supplementary Figure 6. Targeting macrophage CB2 delays tumor progression. Related to Figure 7.

a, CB2 antagonists inhibit 4T-1 tumor growth. Six-week-old mice were subcutaneously inoculated with 4T-1 tumors and treated with CB2 antagonists, AM-630 (0.3 mg $kg^{-1}d^{-1}$, i.p.) or JTE-907 (0.5 mg $kg^{-1}d^{-1}$, i.p.). The tumor volume was measured dynamically. The data represent means ± s.e.ms. (n=10, *P<0.05, Student's *t*-test).

b, CB2 antagonists improve the survival of 4T-1 tumor-bearing mice. The tumor-bearing mice were described in (**a**). The survival time was recorded. The experiment was repeated twice (n=10, **P<0.01, Gehan-Breslow-Wilcoxon test).

c, CB2 antagonists inhibit CT-26 tumor growth. Six-week-old mice were subcutaneously inoculated with CT-26 tumors and treated with CB2 antagonists, AM-630 (0.3 mg $kg^{-1}d^{-1}$, i.p.) or JTE-907 (0.5 mg $kg^{-1}d^{-1}$, i.p.). The tumor volume was measured dynamically. The data represent means ± s.e.ms. (n=10, ***P<0.005, Student's *t*-test).

d, CB2 antagonists improve the survival of CT-26 tumor-bearing mice. The tumor-bearing mice were described in (**c**). The survival time was recorded. The experiment was repeated twice (n=10, *P<0.05, Gehan-Breslow-Wilcoxon test).

e, Immunoblotting assays of MGLL protein in control or CB2-KO MC-38 cells.

f, The inoculated CB2-KO MC-38 tumors were inhibited by CB2 antagonists. Six-week-old WT mice were subcutaneously inoculated with CB2-KO MC-38

cells and treated with CB2 antagonists, AM-630 (0.3 mg $kg^{-1}d^{-1}$, i.p.) or JTE-907 (0.5 mg $kg^{-1}d^{-1}$, i.p.), or PBS as a control. The tumor volume was measured dynamically. The data represent the means±s.e.ms. (n=5, *$P<0.05$, Student's *t*-test).

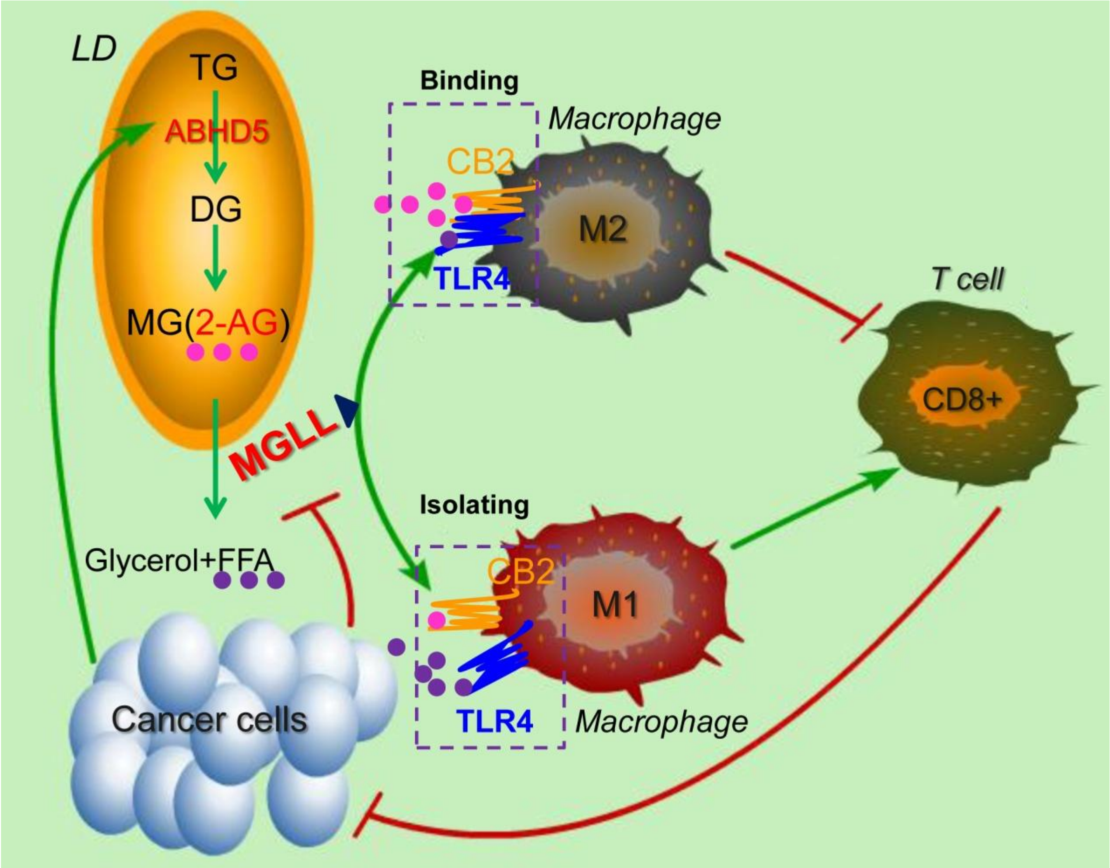

Supplementary Figure 7. MGLL regulates CB2/TLR4-dependent TAM activation and tumor progression

CB2 works as a brake of TLR4, and MGLL functions as a two-way switch in the regulation of macrophage activation via CB2/TLR4 interaction. When macrophage MGLL was upregulated or activated, decrease of 2-AG would discharge the CB2-suppressed TLR4 signal, and simultaneously increased free fatty acid might activate TLR4 signal transduction. When macrophage MGLL was deficient (For example, in tumor microenvironment), increase of 2-AG would activate the combination of CB2 and TLR4, and simultaneously free fatty acid-stimulated TLR4 would also be restricted. The M1 or M2-like TAMs further regulate CD8+ T cell activity and tumor progression. The arrow indicates positive regulation, and the line represents negative regulation. LD, lipid droplet; TG, triglyceride; DG, diglyceride; MG, monoglyceride; 2-AG, 2-arachidonoylglycerol; CB2, cannabinoid receptor 2; TLR4, toll-like receptor 4

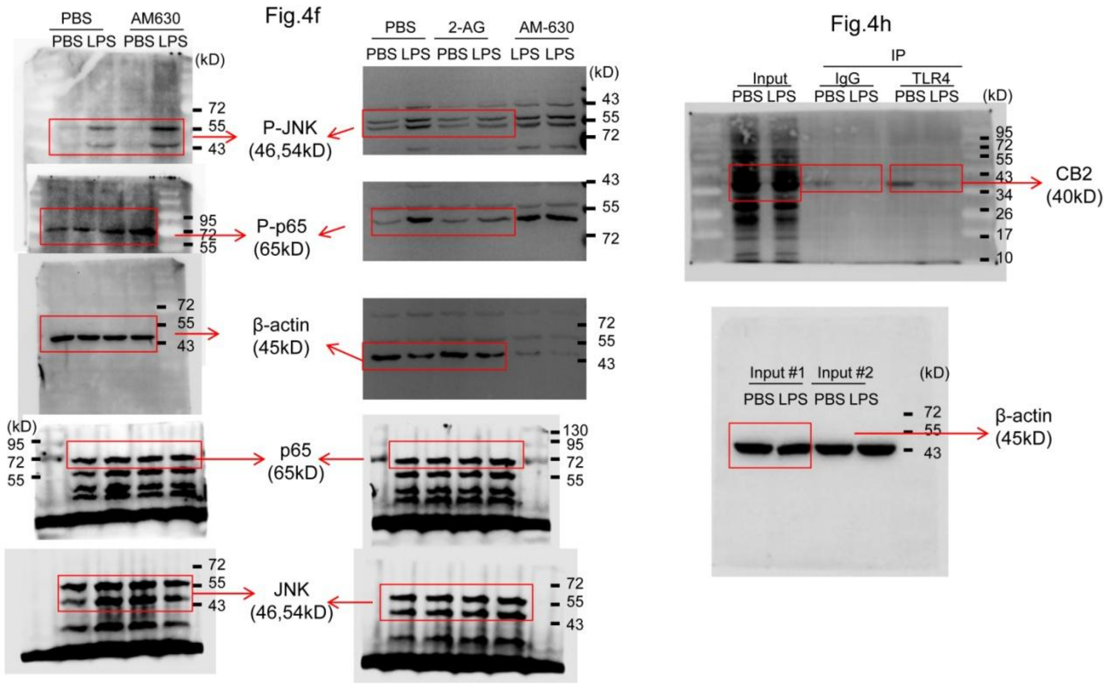

Supplementary Figure 8. The primary images for the cropped blots in Figure 4f and 4h

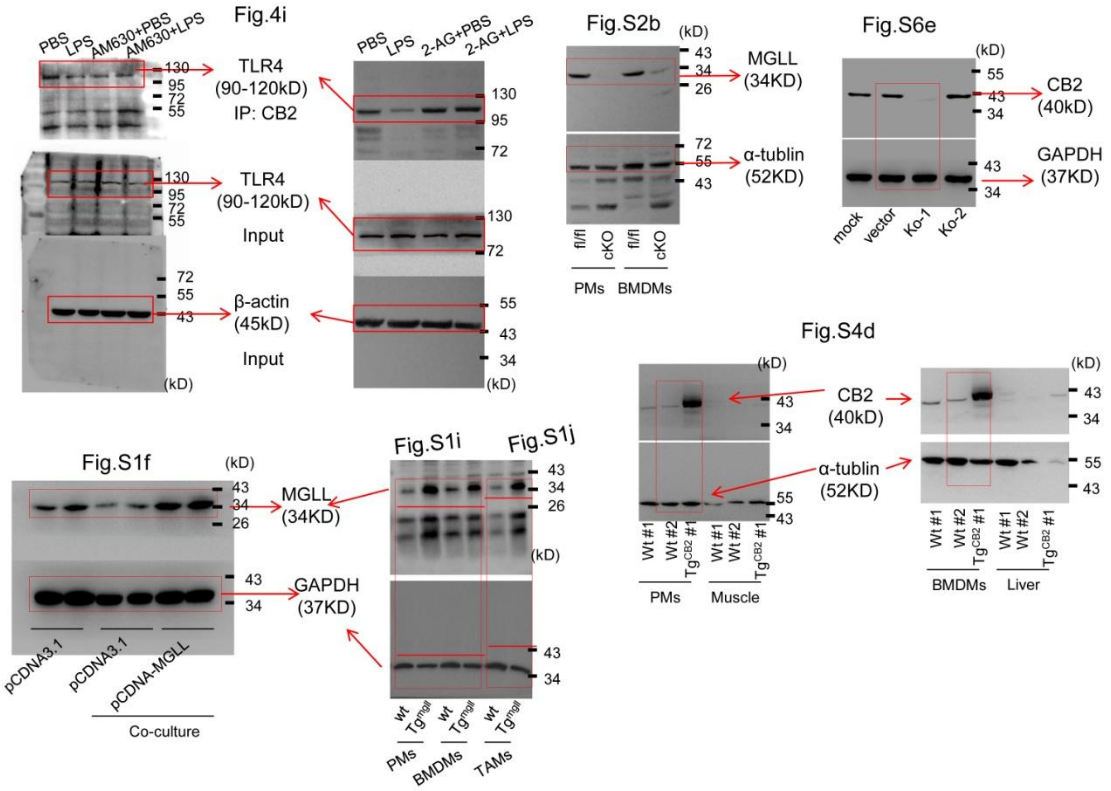

Supplementary Figure 9. The primary images for the cropped blots in Figure 4i and supplementary Figures 1f, 1i, 1j, 2b, 4d and 6e

Supplementary Table 1. Primer sequences for Real-time PCR

Gene	Species	Forward primers（5'→3'）	Reverse primers（5'→3'）
MGLL	mouse	CAGAGAGGCCCACCTACTTTT	ATGCGCCCCAAGGTCATATTT
IL-1β	mouse	ACTCATTGTGGCTGTGGAGA	TTGTTCATCTCGGAGCCTGT
IL-6	mouse	TCGTGGAAATGAGAAAAGAGTTG	AGTGCATCATCGTTGTTCATACA
TNFα	mouse	CTGAGGTCAATCTGCCCAAGTAC	CTTCACAGAGCAATGACTCCAAAG
IL-10	mouse	GAGAAGCATGGCCCAGAAATC	CGCATCCTGAGGGTCTTCA
Arg-1	mouse	CTGAGCTTTGATGTCGACGG	TCCTCTGCTGTCTTCCCAAG
TGF-β	mouse	GACCCTGCCCCTATATTTGGA	CCGGGTTGTGTTGGTTGTAGA
CB2	mouse	CCTGGGATAGCTCGGATGCG	GTGGTTTTCACATCAGCCTCTGTTTC
CB1	mouse	GGGCACCTTCACGGTTCTG	GTGGAAGTCAACAAAGCTGTAGA
FASN	mouse	GCTGCGGAAACTTCAGGAAAT	AGAGACGTGTCACTCCTGGACTT
DGAT1	mouse	AGAAGAGGACGAGGTGCGA	GATGGCACCTCAGATCCCAGTAG
DGAT2	mouse	CATCATCGTGGTGGGAGGTG	TGGGAACCAGATCAGCTCCAT
ABHD5	mouse	TTGCAGGACCTTTTGGGTTA	TCACCGTGTCATCTTCAAACA
ATGL	mouse	GAGAGAACGTCATCATATCCCACTT	CCACAGTACACCGGGATAAATGT
HSL	mouse	GGAGCACTACAAACGCAACGA	TCGGCCACCGGTAAAGAG
ANGPTL4	mouse	GCCTTTCCCTGCCCTTCTC	GATTGGAATGGCTACAGGTACCA
LPL	mouse	ACTCTGTGTCTAACTGCCACTTCAA	ATACATTCCCGTTACCGTCCAT
CD36	mouse	GGAACTGTGGGCTCATTGC	CATGAGAATGCCTCCAAACAC
IFNγ	mouse	CAGCAACAGCAAGGCGAAA	CTGGACCTGTGGGTTGTTGAC
IL-4	mouse	AACGAGGTCACAGGAGAAGG	TCTGCAGCTCCATGAGAACA
β-actin	mouse	AGCCATGTACGTAGCCATCC	CTCTCAGCTGTGGTGGTGAA
MGLL	human	ACAAGTCAGGTCAGGCTTCA	AAGTGGGGCCTTTCATAGCT
CB2	human	ATAGACCTACCATGTGCCGG	GCATCTTTGAGGTTGCCACA
TGF-β	human	TTGAGACTTTTCCGTTGCCG	CGAGGTCTGGGGAAAAGTCT
β-actin	human	GGACTTCGAGCAAGAGATGG	AGCACTGTGTTGGCGTACAG

Supplementary Table 2. Characteristics of patients from whom samples were used for macrophage isolation

Gender	N	Age	Stage	Smoker(%)	Chemotherapy before surgery(%)
Male	44	65.1	$T_{2-4}N_{0-2}M_{0-1}$	86.4	0
Female	62	60.7	$T_{2-4}N_{0-2}M_{0-1}$	37.1	0
